# Highly efficient degradation of pharmaceutical sludge by catalytic wet oxidation using CuO-CeO2/γ-Al2O3 as a catalyst

**DOI:** 10.1371/journal.pone.0199520

**Published:** 2018-10-10

**Authors:** Xu Zeng, Jun Liu, Jianfu Zhao

**Affiliations:** State Key Laboratory of Pollution Control and Resources Reuse, College of Environmental Science and Engineering, Tongji University, Shanghai, China; Babasaheb Bhimrao Ambedkar University, INDIA

## Abstract

Pharmaceutical sludge is considered as a hazardous material with high treatment and disposal costs. In the present study, the catalytic wet oxidation (CWO) of pharmaceutical sludge by CuO-CeO_2_/γ-Al_2_O_3_ as the catalyst was investigated. The catalyst was prepared by traditional wet impregnation. The catalyst was characterized using X-ray Powder Diffraction (XRD) and Scanning Electron Microscopy (SEM). CWO was performed in an experimental batch reactor. Several parameters that could affect the catalytic degradation efficiency, including catalyst dose, temperature, time, oxygen pressure and pH, were investigated. Under optimum conditions, the highest removal rate of volatile suspended solids (VSS) was 87.3% and was achieved at 260°C for 60 min with an oxygen pressure of 1.0 MPa and 10 g/L of catalyst. At the same time, the chemical oxygen demand (COD) removal rate reached as high as 72.6%. This work implies that catalytic wet oxidation is a promising method for the highly efficient degradation of pharmaceutical sludge.

## Introduction

Large volumes of pharmaceutical sludge are produced from pharmaceutical wastewater plants, and they pose containing high environmental risk because of the hazardous and refractory organic pollutants contained in the sludge [1, 2]. In particular, pharmaceutical sludge contains relatively high levels of soluble organics, heavy metals, and recalcitrant antibiotics [3, 4], such as benzylpenicillin [5], aureomycin [6], and berberine hydrochloride [7]. Biological methods are more effective and less costly [8]. However, they are ineffective for removing all potentially hazardous constituents. Physicochemical treatment methods are always complicated [9], while incineration is the most efficient method. Unfortunately, incineration can release noxious compounds (oxides of sulfur and nitrogen, furan) into the air. Pharmaceutical sludge incurs high treatment and disposal costs [10]. Therefore, an economical and green process for the treatment of pharmaceutical sludge is strongly desired.

Advanced Oxidation Processes (AOPs) are promising alternatives for organic waste treatment as they can provide complete mineralization of organic pollutants. Among AOPs, wet air oxidation (WAO) is an attractive technique for industrial organic waste treatment. WAO technology involves oxidation reactions using air or oxygen gas as oxidants under high-temperature and high-pressure conditions [11]. WAO has been proven to be an efficient technology to eliminate highly concentrated, toxic, and hazardous organic compounds. In this process, the pollutants are oxidized by oxygen to carbon dioxide, water, and other products without the emissions of nitrogen oxides, sulfur dioxide, hydrogen chloride, dioxins [12]. Therefore, WAO is an interesting alternative for the solubilisation and mineralization of activated sludge [13–15]. Lab-scale and industrial-scale experiences have confirmed the good performance of WAO on volatile suspended solids (VSS) and chemical oxygen demand (COD) removal [16]. Gasso et al studied the wet oxidation of toxic effluents and organic industrial aqueous wastes [17]. Their results showed that the wet oxidation process using compact jet-mixer reactors is a promising strategy for the treatment of organic aqueous effluents. However, to obtain reasonably high removal and conversion rates, WAO had to be performed under high temperature and high pressure [18].

In catalytic wet air oxidation (CWAO), the addition of catalysts can decrease the operating temperature, enhance the reaction rate, and shorten the residence times [19, 20]. Extensive studies have been conducted on the development of catalysts over the past decades [21, 22]. Among various catalysts, catalysts containing Cu^2+^ or Ce^2+^ have attracted considerable attention due to their good catalytic results for the oxidation of wastewater and sludge. Copper species, which are less pH dependent, have received considerable attention in the last ten years because the redox reaction with Cu species is more stable than with iron [23]. Wang et al. studied the wet air oxidation of pharmaceutical wastewater by a Cu^2+^ and [P_x_W_m_O_y_]^q-^ co-catalyst system. Their results showed that over 40% of COD and TOC removal was obtained at 523 K and 1.4 MPa [24]. The Mn/Ce catalyst presents a better catalytic activity than others in the CWAO of high concentration pharmaceutical wastewater. The biodegradability of poisonous, harmful, and hard-to-degrade organic wastewater can be improved greatly after being treated with CWAO [25]. Mixed Cu^2+^ and Fe^2+^ metallic salts can also act as efficient catalysts. Iron favours the solubilisation of solid organic matter, while copper improves the mineralization of organic compounds in the liquid phase [26]. Zhang et al reported that a Cu–Ni bimetal-based g-Al_2_O_3_/TiO_2_ catalyst for wet oxidation catalysis under microwave irradiation was effectively synthesized via a wet impregnation method. The as-synthesized catalyst had a typical mesoporous structure with a two-dimensional macrostructure which enhanced the catalytic effect [27]. Recently, the development of a novel ceria catalyst has been studies extensively. However, only a few studies have been devoted to real industrial sludge. Therefore, the treatment of real industrial pharmaceutical sludge using the novel catalyst is worthwhile.

In this study, a suitable catalyst for catalytic wet oxidation of pharmaceutical sludge is synthesized. The effects of the oxidation conditions (i.e., catalyst dose, temperature, time, oxygen gas pressure, and pH) were also discussed. The goal of this work is to assess the degradation efficiency of pharmaceutical sludge by catalytic wet oxidation.

## Materials and methods

### Materials

The experiments were performed with pharmaceutical sludge obtained from a pharmaceutical factory in Taizhou (China). The sludge was extracted from a thickening unit, and stored at 4°C until further usage. The initial characteristics of the sludge were the following: the mixed liquor COD was 15 000~16 000 mg/L, the VSS was 13.5 ~ 13.8 g/L, the SS was 15.8 ~ 16.5 g/L, and the pH was 7.5~8.0. The chemical composition of pharmaceutical sludge includes: protein 0.035~0.038 g/L, polysaccharides 0.321~0.342 g/L. The materials used in these experiments, such as Cu(NO_3_)_2_, Ce(NO_3_)_3_, CoO, Fe_2_O_3_, NaOH and HCl, etc, were purchased from Sinopharm Chemical Reagent, China. All reagents used in this study were analytical reagents (≥99%) and were used as received without further purification. The gaseous oxygen used as the oxidant was commercial industrial gas.

### Catalyst

The supported catalyst was synthesized by a typical wet impregnation procedure. First, Cu(NO_3_)_2_ and Ce(NO_3_)_3_ were mixed in a 1:1 molar ratio, where the concentration was 1.0 mol/L. Then, γ-Al_2_O_3_, as a carrier, was placed in the liquor, which was dipped for 24 h. After impregnation, the material was baked for 2.5 h at 550°C to prepare the CuO-CeO_2_/γ-Al_2_O_3_ catalyst.

### Experimental procedure

All experiments were conducted in an SUS316 reactor, purchased from Anhui Kemi Machinery Technology Co. Ltd, China. The internal volume of the reactor was 100 mL. The typical procedure is as follows: desired amounts of the synthetic pharmaceutical sludge, catalyst and oxygen were put into the reactor, and the temperature was then increased to 180–260°C. After the desired reaction temperature was achieved, the reaction time was started. Once the desired reaction time elapsed, the reactor was removed from the oven and allowed to cool to room temperature. Generally, approximately 30 min was required to heat the liquid to their reaction temperature in the reactors, and the pressure in the reactor corresponded to the saturated vapour pressure of water. When the liquid was cooled to room temperature, it was sampled and analyzed.

### Analysis methods

The surface morphology of the prepared catalysts and the carrier were surveyed by scanning electron microscopy (Agilent 8500 FE-SEM). The elemental content of the catalysts was analysed by X-ray diffraction (XRD-7000, Japan), using a Siemens model with Cu-Kα radiation.

The pH was measured by a pH meter (pH-201, Hanna Corporation, Italy). Since the composition of the synthetic pharmaceutical wastewater is very complex, COD was used to assess the treatment efficiency in this study, which was measured by the potassium dichromate oxidation method (Hach Heating System, Hach Corporation, USA). Sludge VSS was measured by the ignition loss method.

## Results and discussion

### Catalyst characterization

**Fig 1:**
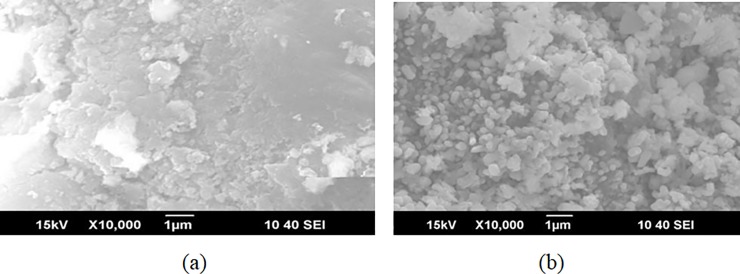
SEM images of catalysts before (a) and after (b) loading.

**Fig 2:**
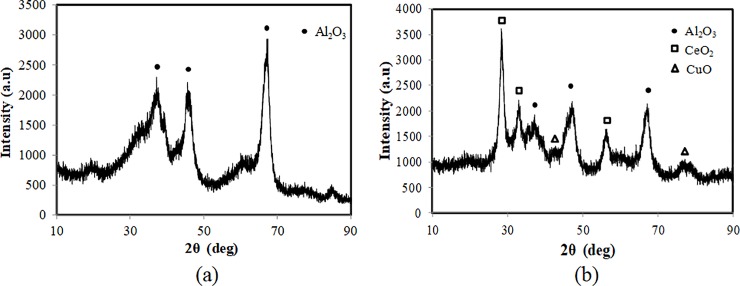
X-ray diffraction patterns of catalysts before (a) and after (b) loading.

Fig 1 shows the SEM images before and after loading the active ingredients on the γ-Al_2_O_3_, and many uniform particles can be seen on the surface of the γ-Al_2_O_3_. These particles were packed closely on the surface of the carrier, which ensures that the active components produce their full catalytic effect, and the retained Cu^2+^ was lost from the surface of the support. The XRD patterns of the solid products before and after loading are given in Fig 2. As shown in Fig 2, it was very clear that new peaks were detected after loading. Before loading, the XRD pattern showed only γ-Al_2_O_3_. After loading, new peaks from the loaded CuO-CeO_2_ were clearly visible. Then, catalytic wet oxidation of the sludge was performed with the synthesized catalyst.

### Catalytic wet oxidation experiments

**Fig 3:**
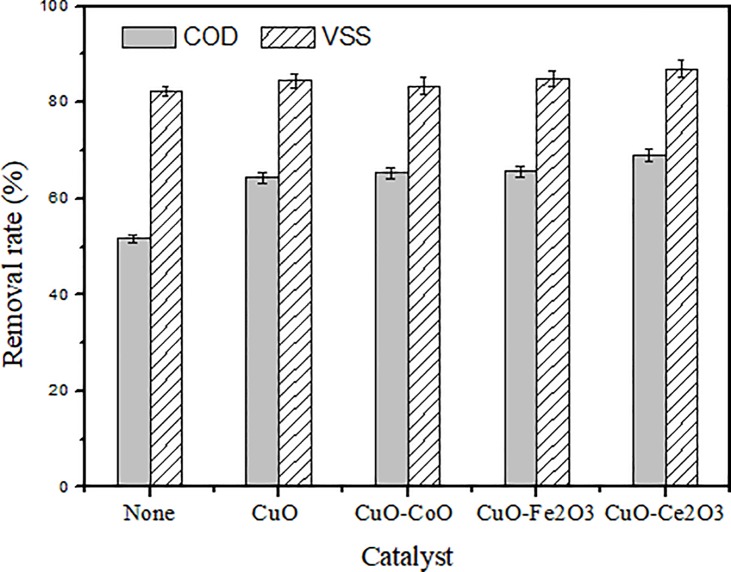
Effect of catalyst (Temp. 240°C, time 60 min, initial oxygen supply 1.0 MPa).

First, different catalysts were analysed to select the most suitable catalyst. The COD and VSS removal rates for different catalysts at a residence time of 60 min under oxygen pressure are described in Fig 3. The activity of these catalysts for the conversion of sludge was clearly visible and the best results were shown for the CuO-CeO_2_ catalyst. The removal rate of COD and VSS reached 69.3% and 86.8%, respectively, which was an increase of 18% and 5% compared with that without the catalyst. CuO as the active component of the catalyst showed diminished results; the removal rate of COD and VSS increased by 13% and 2%. Ce is a rare-earth element that is widely used in catalytic wet oxidation, and its excellent oxygen storage capacity can stabilize the crystal structure and prevent volume contraction [28], which allows the CuO/CeO_2_ catalyst to exhibit catalytic activity in this environment.

**Fig 4:**
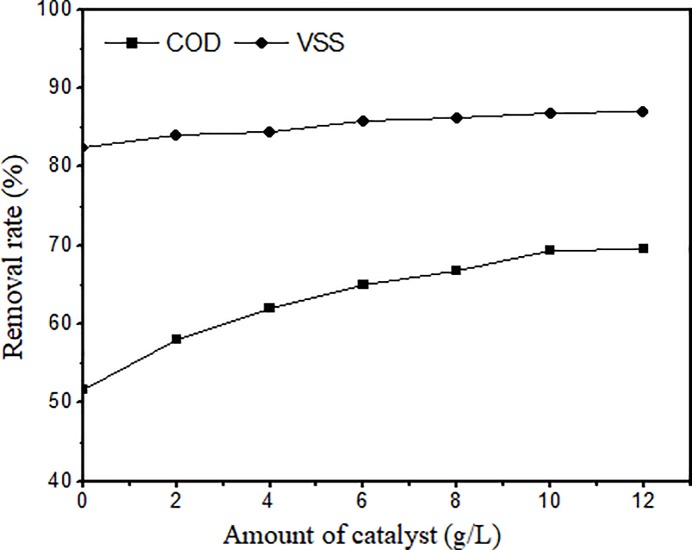
Effect of catalyst dose (Temp. 240°C, time 60 min, initial oxygen supply 1.0 MPa).

The catalyst dose is an extremely important factor for the catalytic wet oxidation to form strongly oxidizing hydroxyl radicals. The effect of the catalyst dose on the COD and VSS removal rates from pharmaceutical sludge was investigated, as shown in Fig 4. It was concluded that the VSS and COD removal efficiency were strongly linked with catalyst dose. The amount of the catalyst added in CuO-CeO_2_/γ-Al_2_O_3_ was increased from 0 to 12 g/L. Fig 4 shows that as more catalyst was added, the COD removal rate first increased and then remained unchanged. Without the catalyst, the COD removal rate was approximately 51%. When the amount of catalyst was 10 g/L, the highest COD removal rate was 69.3%, which was an increase of 18%. However, when the amount of catalyst was increased to 12 g/L, the COD removal rate did not increase. The sludge VSS removal rate increased with the amount of the catalyst, but the influence was not significant. In particular, after the addition of 8 g/L catalyst, there was no improvement. Considering the catalytic effect and dissolution of Cu^2+^, the catalyst dose was chosen as 10 g/L in this experiment.

**Fig 5:**
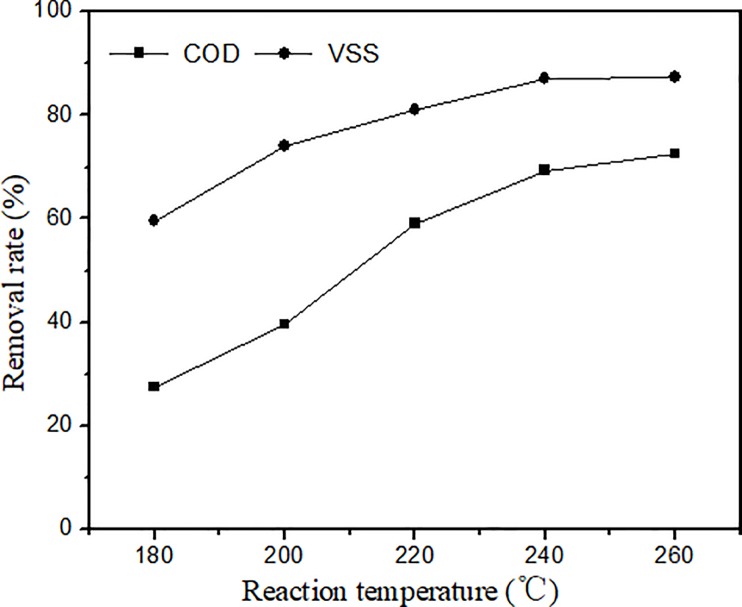
Effect of temperature (time 60 min, initial oxygen supply 1.0MPa, catalyst 10 g/L).

The effect of the reaction temperature (in the range of 180–260°C) on the COD and VSS removal rates from pharmaceutical sludge at a residence time of 60 min under oxygen pressure was investigated. The results can be seen in Fig 5. During CWAO, the COD and VSS removal rates increased as the temperature increased. The effect of the reaction temperature on the catalytic wet oxidation is also very significant. When the reaction temperature was 180°C, the COD and VSS removal rates were 27.4% and 51.6%. When it was 240°C, the COD and VSS removal rates reached 69.3% and 87.1%. At 260°C, the COD and VSS removal rates reached 72.6% and 87.3%. When the temperature was increased from 180 to 240°C, the removal rates of COD and VSS increased significantly, but the improvement was not obvious from 240 to 260°C. This is probably because of the incomplete degradation of COD, because even the organic pollutants in the sludge were almost completely oxidized. It has been reported that the products of the wet oxidation of organics are mainly carboxylic acids with small molecular weights, such as formic acid or acetic acid, which are not easily oxidized under hydrothermal conditions. From the above results, we conclude that higher temperatures were favourable for the CWAO of pharmaceutical sludge. However, from a practical point of view, higher temperatures lead to higher operating costs and more severe corrosion problems. Therefore, the reaction temperature of 240°C was adopted in the following experiments examining other operating parameters.

**Fig 6:**
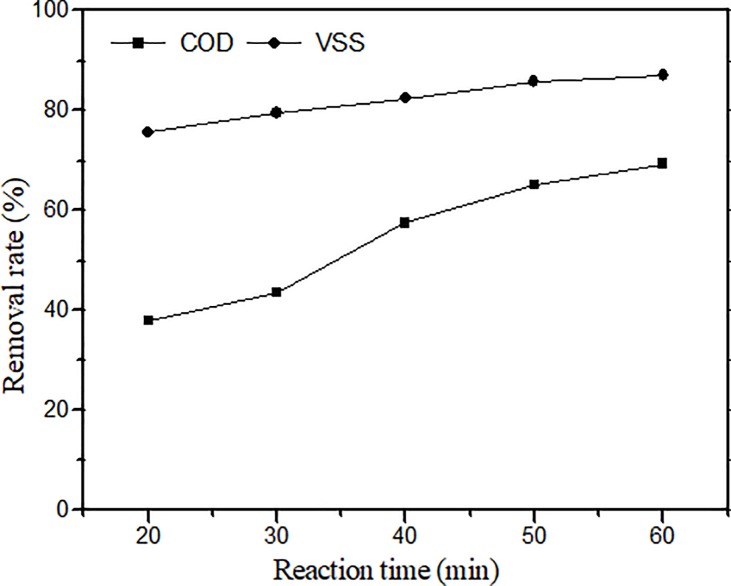
Effect of reaction time (Temp. 240°C, initial oxygen supply 1.0MPa, catalyst 10g/L).

Fig 6 shows the effect of reaction time on the COD and VSS removal rates during CWAO at 240°C with an oxygen pressure of 1.0 MPa and 10 g/L of the catalyst. The reaction time was changed from 20 min to 60 min. The results show that in catalytic wet oxidation, the reaction time affects the sludge treatment. The removal of VSS from the sludge was 75.6% after 20 min, and most VSS was degraded in a relatively short period of time. As the time increased, the removal rate gradually increased. The final VSS removal rate increased by approximately 5% compared with the rate of the catalyst. The COD removal rate also increased with reaction time. However in the initial stage, the COD removal rate did not increase, whereas after 20–30 min, the COD removal rate greatly improved. The rate was the highest in the whole process. As the time continued to increase, the removal rate first grew but then began to decrease. At 60 min, the COD removal rate reached 69.3%, compared with the rate of the catalyst of 17.6%. These phenomena indicate that in the catalytic wet oxidation process, the organic matter with low activation can participate in the reaction first without the catalyst and rapid oxidation. As the time was increased, solid-phase organic matter transferred into the liquid phase, and the degradable organic matter accumulated in the liquid due to the presence of the catalyst. Organic matter with higher activation energy began to oxidize, and the overall reaction rate greatly improved. Then, as the concentration of the reactants decreased, the reaction rate decreased. Considering the oxidation efficiency and the economic factors, we chose 60 min as the reaction time for the subsequent study of catalytic wet air oxidation.

**Fig 7:**
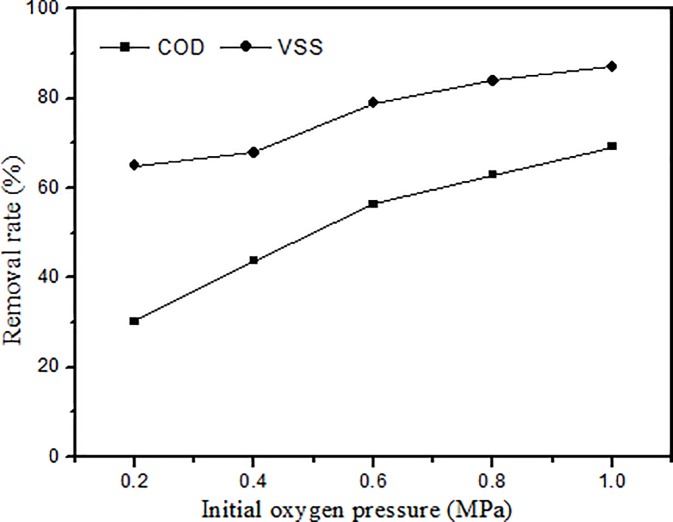
Effect of initial oxygen supply (Temp. 240°C, time 60min, catalyst 10g/L).

To gain better insight into the effect of oxygen pressure on the COD and VSS removal rates, experiments were conducted under initial oxygen pressures varying from 0.2 to 1.0 MPa. The results are shown in Fig 7. In the treatment of pharmaceutical sludge by catalytic wet oxidation, the initial oxygen pressure greatly impacts the COD and VSS removal rates. With increased oxygen pressure, the COD and VSS removal rates gradually increased. The VSS removal rate was affected more by the initial oxygen pressure; when the oxygen pressure was 0.2 MPa, the removal rate of VSS was 65%, and when the pressure rose to 1.0 MPa, the removal rate increased by 22.1%. The increase occurred primarily between 0.4 and 0.6 MPa. Moreover, the presence of the catalyst to some extent changed the VSS removal reaction process. The removal rate of COD greatly increased as the pressure was increased from 0.2 to 0.6 MPa. As the pressure continued to increase, the removal rate of COD increased, but the rate of increase slowed. The catalyst changed the oxygen utilization rate. COD removal was also obviously increased at a low initial oxygen pressure. Under higher oxygen pressures, the dissolved oxygen concentration increased and favoured the formation of strong oxidative species, which resulted in enhanced oxidation. Therefore, the high pressure could efficiently accelerate the oxidation reaction rate and eliminate organic compounds, resulting in the achievement of high COD and VSS removal [29].

The real pharmaceutical sludge had a neutral pH. The effect of pH on the COD and VSS removal rates was studied under different pH conditions: acidic and alkaline. The results in Table 1 show that the COD and VSS removal rates were similar under different pH conditions, which means that pH adjustment is not necessary for the catalytic wet oxidation of this kind pharmaceutical sludge.

**Table 1:** Effect of pH value on CWAO of pharmaceutical sludge*.

pH value	COD removal rate (%)	VSS removal rate (%)
5.12	65.9	54.8
8.08	69.3	86.8
11.05	61.2	86.7

*Reaction condition: Temp. 240°C, 60min, oxygen pressure 1.0 MPa, catalyst 10g/L.

## Conclusions

In this study, the catalytic wet oxidation of pharmaceutical sludge by a prepared catalyst was studied. The catalyst was prepared by traditional wet impregnation. The catalyst was characterized using XRD and SEM. CWO was performed in an experimental batch reactor. Several parameters that could affect the catalytic degradation efficiency, including catalyst dose, temperature, time, oxygen pressure and pH, were investigated. The VSS in pharmaceutical sludge were significantly reduced. Under optimum conditions, the highest removal rate for VSS was 87.3% and was achieved at 260°C over 60 min with an oxygen pressure 1.0 MPa and 10 g/L of catalyst. At the same time, the COD removal rate reached as high as 72.6%. This work implies that catalytic wet oxidation is a promising method for the highly efficient degradation of pharmaceutical sludge.
